# COVID19: A Systematic Approach to Early Identification and Healthcare Worker Protection

**DOI:** 10.3389/fpubh.2020.00205

**Published:** 2020-05-19

**Authors:** Yu Zhao, Chong Cui, Kun Zhang, Jialin Liu, Jinfu Xu, Eric Nisenbaum, Yixiang Huang, Guoyou Qin, Bing Chen, Michael Hoffer, Susan H. Blanton, Fred Telischi, Joshua M. Hare, Sylvia Daunert, Bhavarth Shukla, Savita G. Pahwa, Dushyantha T. Jayaweera, Paul E. Farmer, Carlos del Rio, Xuezhong Liu, Yilai Shu

**Affiliations:** ^1^ENT Institute and Otorhinolaryngology Department of the Affiliated Eye and ENT Hospital, State Key Laboratory of Medical Neurobiology, Institutes of Biomedical Sciences, Fudan University, Shanghai, China; ^2^Department of Critical Care Medicine, Ruijin Hospital, Shanghai Jiao Tong University School of Medicine, Shanghai, China; ^3^Department of Respiratory and Critical Care Medicine, Shanghai Pulmonary Hospital, Tongji University School of Medicine, Shanghai, China; ^4^Department of Otolaryngology, University of Miami Miller School of Medicine, Miami, FL, United States; ^5^Department of Health Policy and Management, Health Development Research Center, School of Public Health, Sun Yat-Sen University, Guangzhou, China; ^6^Department of Biostatistics, School of Public Health, and the Key Laboratory of Public Health Safety of Ministry of Education, Fudan University, Shanghai, China; ^7^Department of Neurological Surgery, University of Miami Miller School of Medicine, Miami, FL, United States; ^8^Dr. John T. Macdonald Foundation, Department of Human Genetics and John P. Hussman Institute for Human Genomics, University of Miami Miller School of Medicine, Miami, FL, United States; ^9^Department of Biochemistry and Molecular Biology, University of Miami Miller School of Medicine, Miami, FL, United States; ^10^Division of Infectious Diseases, Department of Medicine, University of Miami Miller School of Medicine, Miami, FL, United States; ^11^Partners in Health, Boston, MA, United States; ^12^Harvard Medical School, Boston, MA, United States; ^13^Brigham & Women's Hospital, Boston, MA, United States; ^14^Division of Infectious Diseases, Department of Internal Medicine, Emory University School of Medicine, Atlanta, GA, United States

**Keywords:** COVID-19, SARS—CoV-2, Coronavirus (CoV), healthcare worker protection, COVID 2019

## Abstract

The COVID-19 outbreak spread rapidly throughout the globe, with worldwide infections and deaths continuing to increase dramatically. To control disease spread and protect healthcare workers, accurate information is necessary. We searched PubMed and Google Scholar for studies published from December 2019 to March 31, 2020 with the terms “COVID-19,” “2019-nCoV,” “SARS-CoV-2,” or “Novel Coronavirus Pneumonia.” The main symptoms of COVID-19 are fever (83–98.6%), cough (59.4–82%), and fatigue (38.1–69.6%). However, only 43.8% of patients have fever early in the disease course, despite still being infectious. These patients may present to clinics lacking proper precautions, leading to nosocomial transmission, and infection of workers. Potential COVID-19 cases must be identified early to initiate proper triage and distinguish them quickly from similar infections. Early identification, accurate triage, and standardized personal protection protocols can reduce the risk of cross infection. Containing disease spread will require protecting healthcare workers.

## Introduction

**Figure 1:**
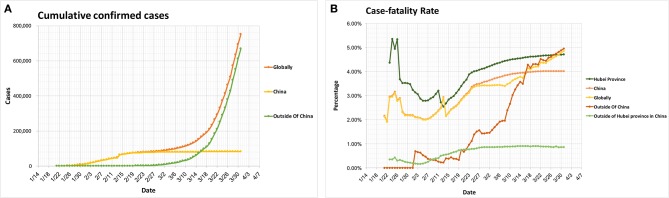
Global epidemiology of COVID-19 cases. **(A)** Cumulative confirmed cases. **(B)** Cases-fatality rate. The case-fatality rate: globally 4.85%, China 4.01%, Outside of China 4.95%, Hubei Province 4.71%, Outside of Hubei province in China 0.86%. All the data comes from the WHO Coronavirus disease 2019 (COVID-19) Situation Report (Data as reported by national authorities by March 31, 2020), except the data in **(B)**, Hubei Province and Outside of Hubei Province in China, which comes from the latest development of the COVID-19 epidemic: General Office of the National Health Council, China.

**Figure 2:**
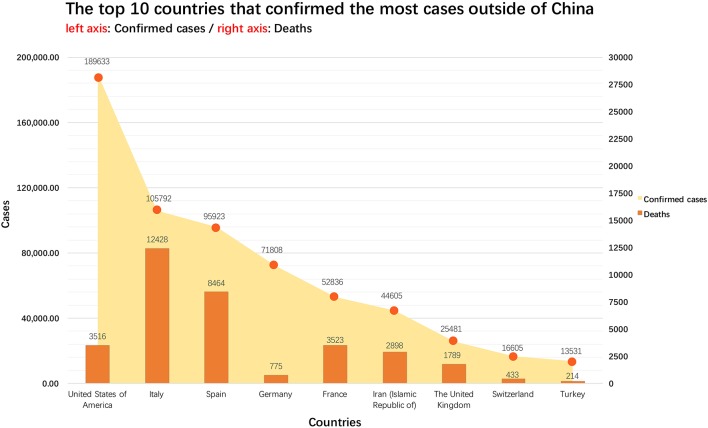
The 10 non-China countries with the most reported cases as of April 1, 2020. All the data comes from Coronavirus COVID-19 Global Cases by Johns Hopkins CSSE (https://gisanddata.maps.arcgis.com/apps/opsdashboard/index.html#/bda7594740fd40299423467b48e9ecf6).

A new coronavirus, SARS-CoV-2, causes coronavirus disease 2019 (COVID-19), with worldwide cases of transmission occurring shortly after the initial infections were reported (1, 2). On January 30, 2020, the International Health Regulations Emergency Committee of the World Health Organization (WHO) declared the outbreak a “public health emergency of international concern (PHEIC),” and on March 11th the WHO officially designated the outbreak as a pandemic. By March 31, 2020, all continents had reported confirmed cases of COVID-19. The global spread of the virus is ongoing and fast-moving (Figure 1A), with over 750,890 confirmed infections and 36,405 deaths worldwide. The global cases-fatality rate is 4.85% with differences in different countries and areas, suggesting that reasonable and effective medical interventions can have a great impact (Figures 1B, 2). Worryingly, COVID-19 cases have now been identified in a number of countries with lower access to health care resources, including 39 African countries, and others (3). Another concern is potential spread among healthcare workers, who by nature of their professions are at an especially high risk of exposure. In China, more than 3,000 health care workers in Hubei Province have been infected, with most being doctors from non-infectious disease departments (4). A total of 34 confirmed deaths among infected healthcare workers in China have been identified as of March 2, 2020 (5). Per the Italian National Health Agency, 9,512 healthcare works have been infected as of March 31th, 2020.

Identifying cases of COVID-19 and controlling its spread can be difficult, as SARS-CoV-2 is infectious during the incubation period (6, 7). Latency from infection to disease manifestation varies from 1 to 14 days, and no or atypical symptoms may appear in its early stages (8, 9). It can be easily confused with other common diseases in its early stage, leading to misdiagnosis or missed diagnosis and subsequent nosocomial spread of disease. This is a major reason for the large number of infections among healthcare workers in both Italy and the city of Wuhan. Therefore, the early identification of COVID-19 patients is critical. At the same time, controlling COVID-19 requires the joint efforts of multiple clinical departments. Accordingly, it is very important to protect healthcare workers, with different levels of standardized protective measures utilized commensurate to exposure risk.

We descriptively reviewed the English and Chinese literature from December 2019 through to March 31st, 2020. The PubMed and Google Scholar databases were searched with the terms “COVID-19,” “2019-nCoV,” “SARS-CoV-2,” and “Novel Coronavirus Pneumonia” in order to identify studies for inclusion. The purpose of this review is to provide current information on the characteristics of SARS-CoV-2 infection and standardized screening, triage, and protection protocols, in order to ensure the early identification of COVID-19 and protection of healthcare workers.

## The Virus and its Pathophysiology

The exact origin of the SARS-CoV-2 has not yet been established. Possibilities include a bat coronavirus termed BatCoV RaTG13, which shares 96.2% of its genome sequence with SARS-CoV-2, though two other similar bat-derived coronaviruses have also been identified (10). However, no evidence of direct bat to human transmission has been reported, leading to the suggestion that minks, pangolins, snakes, and other wild animals may serve as candidate intermediate hosts to transmit the virus to humans (11–13).

SARS-CoV-2 is a single positive strand RNA, ~60–140 nm in diameter. SARS-CoV-2 has 94.6% key region (ORF1ab) homology with SARS-CoV, indicating the two derive from the same species (10). However, the S protein of SARS-CoV-2 binds with human ACE2 with a much higher affinity than the S protein of SARS-CoV, the main reason for the strong infectivity of the new coronavirus (14, 15). In addition, research findings suggest that SARS-CoV-2 invaded host cells via a novel route of CD147-spike protein (SP) (16). This possible mechanism for the entry of SARS-CoV-2 into the cells may provide important information for vaccine development and targeted drug research (15).

## Epidemiological Characteristics

Based on analysis of data from a large number of clinical cases, the median incubation period of COVID-19 is 4–6.4 days, with a basic reproductive number (*R*_0_) of 2.2–2.68 (17, 18).

**Figure 3:**
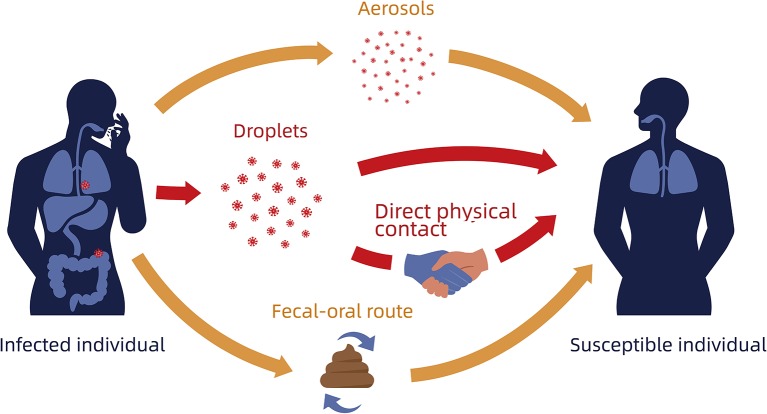
Schematic diagram of SARS-CoV-2 transmission. Red arrows indicate most common transmission; Orange arrows indicate possible routes of transmission.

In addition to patients demonstrating the full COVID-19 disease phenotype, mildly symptomatic and asymptomatic patients are also a source of infection (6). SARS-CoV-2 is spread through respiratory transmission via exhaled droplets (10). Touching the mouth, nose, or eyes after contact with virus-contaminated materials may lead to COVID-19 transmission (19). There is a possibility of airborne transmission when exposed to high concentrations of the aerosolized virus in a relatively closed environment for an extended time (Figure 3) (20). SARS-CoV-2 transmission through the digestive tract may also be possible, but needs further confirmation (21). Compared to SARS, which only affects the respiratory tract, SARS-CoV-2 can multiply effectively in the nose and throat (7, 22). Recent data provides no evidence for intrauterine vertical transmission in pregnant women who are infected with COVID-19 (23).

## Clinical Presentation

Typical clinical symptoms of COVID-19 are fever (83–98.6%) (8, 17, 24, 25), cough (59.4–82%) (8, 17, 24, 25), and fatigue (38.1–69.6%) (8, 17, 24). However, clinical data suggests that only 43.8% of patients have fever at the early stages of the SARS-CoV-2 infection (17), and some patients presented with uncommon symptoms such as increased sputum (15.4–33.7%) (8, 17, 24, 25), shortness of breath (18.7–31%) (17, 25), sore throat (5–17.4%) (8, 17, 25), nasal congestion (4.8%) (17), dizziness (9.4%) (8), and diarrhea (2–10.1%) (8, 17, 24, 25).

Patients with a mild form of the disease presented with only a low fever, mild fatigue, and no pneumonia, with some patients demonstrating no obvious fever throughout the course of the disease. This makes clinical diagnosis difficult in this patient population. However, 7.3–32% of patients progress to a severe (dyspnea, respiratory frequency ≥30/min, blood oxygen saturation ≤93%, partial pressure of arterial oxygen to fraction of inspired oxygen ratio ≤300 mmHg or lung infiltrates >50% within 24–48 h) or critical (respiratory failure, septic shock, and/or multiple organ dysfunction or failure) condition (8, 9, 17, 24, 26). The median time from onset of symptoms to ICU admission was 9.5–10.5 days (8, 24, 26).

According to our experience treating cases in Wuhan, severe and critically ill patients demonstrate the following patterns: (1) Clinical deterioration happens suddenly and is difficult to predict. Acute respiratory failure, especially acute respiratory distress syndrome (ARDS), is very common in COVID-19 severe and critically ill patients. However, compared to previous severe infectious diseases such as SARS or MERS**—**where ARDS often appeared within 1 week of symptom onset**—**patients with COVID-19 showed greater variance in timing. Many patients were stable for more than a week and even up to a month before acute respiratory failure or ARDS suddenly occurred within a 2–3 days period. This makes it difficult for clinicians to predict critical changes in a patient's condition. Once such patients have respiratory failure, they require a prompt upgrade in respiratory support and close observation in order to continue to escalate treatment as necessary. The mortality rate of all COVID-19 patients with ARDS is close to 50%, and if ARDS reaches the moderate to severe stages, the mortality rate is as high as 70%; (27) (2) Multisystem organ failure. From the current epidemiological data, the majority of middle-aged and elderly patients with COVID-19 are critically ill (8, 17, 26) and dysfunction of multiple organ systems including respiratory failure, heart failure, and renal failure can occur either individually at different time points or simultaneously (8, 9, 25, 26). Therefore, it is necessary to frequently and comprehensively evaluate these critically ill patients, with attention paid to each individual organ system; and (3) Multiple comorbidities often exist in severe patients. Multiple chronic diseases such as diabetes, hypertension, CKD, and Parkinson's are frequently found in severe or critically ill patients (8, 9, 17, 26). These diseases often contribute to the course of COVID-19. For example, diabetic patients are at high risk for ketoacidosis during COVID-19 infection, CKD patients often have further deterioration of renal function requiring RRT treatment, and neurological diseases such as Parkinson's compromise airway protection and the ability for patients to cooperate with treatment. Therefore, clinicians need to closely evaluate patients' comorbidities and provide additional disease-specific care while diagnosing and treating COVID-19.

On laboratory testing, peripheral blood leukocyte counts are low or normal and lymphocyte counts are low. Severe and critically ill patients often have elevated inflammatory factors. In early CT chest imaging, there were multiple small patchy and interstitial changes, which then develop into multiple ground glass opacities and infiltrates in the lungs consistent with viral pneumonia. In severe cases, pulmonary consolidation can occur, but pleural effusions are rare.

## Early Disease Identification

The clinical manifestations and illness onset of COVID-19 overlap with or resemble the clinical manifestations of many other diseases, including the common cold, influenza, and other upper respiratory infections (URIs) (Table 1). Some patients who have atypical symptoms or no fever may present to non-infectious disease departments, which may lead to missed or delayed diagnosis, healthcare worker infection, and even nosocomial infections in other patients. The CDC has published primers, probes, and testing protocols, but there were initially issues with the test kit's performance and distribution that prevented a scaling up of testing beyond a few public health laboratories (28). After corrections, the CDC re-released testing protocols that are now being utilized by state and local health departments. The differential diagnosis at the onset of symptoms mainly depends on epidemiological history, complaints, symptoms, and corresponding examinations. Therefore, maintaining a high level of suspicion for early clinical manifestations of COVID-19 and using existing testing to rule out other respiratory viruses is necessary to ensure that patients receive appropriate early molecular testing.

**Table 1:** Major differential diagnosis of COVID-19.

**Disease**	**COVID-19**	**Common Cold**	**Influenza**	**Other URIs**
Etiology	SARS-CoV-2	Common respiratory viruses	Influenza virus	Bacteria/viruses
Illness onset characteristics	Incubation period is 1–14 days, mean 4– 6.4 days	Year round	Year round, high in winter and spring	Year round
Fever characteristics	Fever common on admission	1–2 days, mostly low fever	3–5 days, may be high fever	May be high fever
Clinical symptoms	Typical: Fever, dry cough, and fatigue Atypical: congestion, rhinorrhea, sore throat, myalgia, and diarrhea	Nasal congestion, rhinorrhea, sneezing, sore throat; few systemic symptoms	Severe systemic symptoms such as fever, headache, myalgia, chills, shivering	Nasal congestion, sore throat, swallowing pain, cough; systemic symptoms such as fever, fatigue/malaise
Infectivity	Strong, potential for pandemic	Weak, mostly distributed	Strong, potential for pandemic	Weak, mostly distributed
Complication	Septic shock, ARDS, acute kidney injury, disseminated intravascular coagulation, rhabdomyolysis	Rare	Pneumonia, otitis media, myocarditis, rhabdomyolysis, septic shock.	Acute sinusitis, acute otitis media, acute pharyngitis, acute bronchitis, pneumonia.
Diagnosis	Epidemiology, clinical symptoms, viral nucleic acid test, serological examination (IgM/IgG)	Clinical symptoms	Respiratory tract virus nucleic acid detection	Clinical symptoms

## Outpatient and Emergency Pre-Check and Triage

**Figure 4:**
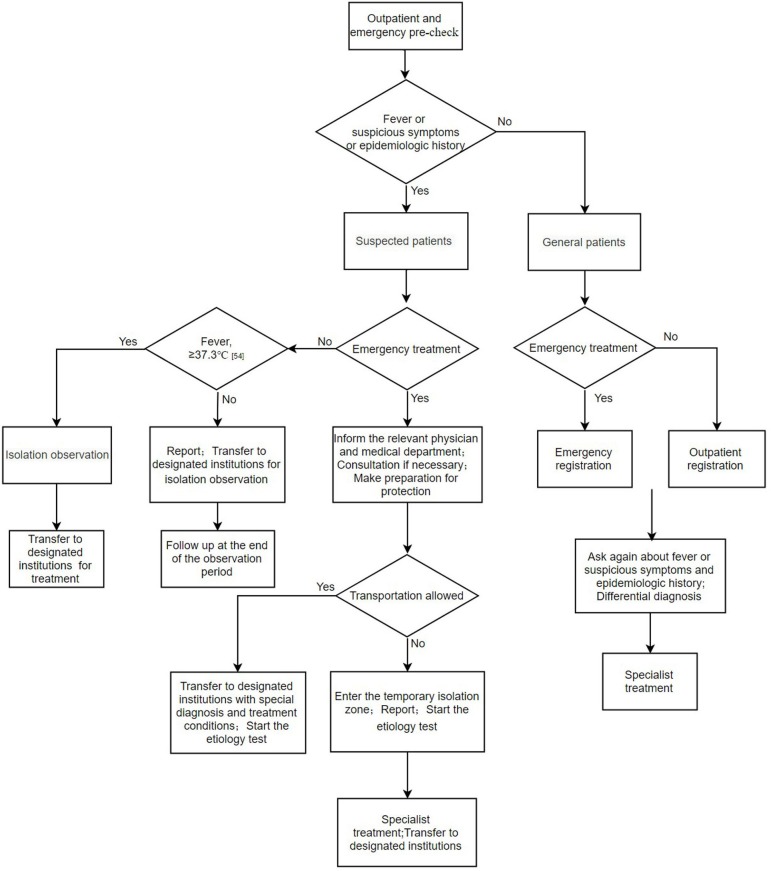
Suggested strategy of outpatient and emergency triage flow chart.

Some patients with atypical symptoms or no fever may initially present to non-infectious disease departments such as otolaryngology, stomatology, and gastroenterology which are not standardly equipped to safely handle these patients. In addition, many countries do not have a hierarchical medical system; in such countries patients decide on their own as to which hospitals and departments to present. This poses considerable risks to healthcare workers and other patients in these hospitals. Successful disease control requires a systematic, comprehensive approach to diagnosis, triage, and treatment. Outpatient and emergency pre-check is the first step to prevent and control the potential spread of COVID-19 in healthcare settings. By initially screening patients by obtaining a temperature, inquiring about the epidemiological history, and triaging according to predetermined symptoms, suspected cases can be identified. Suspected cases should be isolated and reported immediately. In addition, medical staff, in consultation with local public health authorities, should collect the necessary specimens (for example nasopharyngeal swab) for COVID-19 nucleic acid detection in a timely fashion. If the patient's condition requires supportive care in an intensive care unit, he or she should be placed in an isolation negative pressure room. If such an area is not available, then the patient should be transferred to a facility that can provide this care after the patient's condition is stabilized (Figure 4) (29).

## Protection of Healthcare Workers

Designated institutions, such as fever clinics in China and infectious disease departments in other countries, have been established in order to ensure routine and appropriate infection control operations for COVID-19 cases. However, in healthcare settings where patients with emerging or unknown infectious diseases are diagnosed and treated, especially in those countries where there is no designated institution for COVID-19 cases, infection control methods against severe infectious diseases are often imperfect. With the outbreak of COVID-19, healthcare workers are at particular risk for infection due to their potential exposure to droplets or aerosols from the respiratory tracts of patients.

For providing clinical care to patients with COVID-19, all healthcare workers are recommended to receive standardized infection prevention and control (IPC) training and wear correct personal protective equipment (PPE) (e.g., medical masks such as surgical masks or N-95 respirators depending on exposure risk, disposable isolation gowns, disposable patient examination gloves, working hat, goggles or face shield, and shoe covers) (30).

In the epidemic area, considering it is not always possible to identify patients with SARS-CoV-2 infection because early symptoms are non-specific, it is important that healthcare workers use standard precautions consistently when providing care to patients. Refresher training on IPC should be provided to every healthcare worker (31), healthcare workers should practice donning and doffing procedures, and must demonstrate competency through testing and assessment before caring for patients (32). This is particularly true of frontline providers such as those in the infectious disease and emergency departments, as well as providers in departments who may have higher occupational exposure risks when conducting special examinations on patients, such as the departments of Otorhinolaryngology, Stomatology, Gastroenterology, and Pulmonology, among others. Healthcare workers in these departments should pay extra attention to proper protection, and specialty or exam specific protective protocols should be developed (10). For example, nasopharyngeal swab specimen collection is a common and simple disease detection method, but it has certain exposure risks such as causing patients to sneeze. It must be performed in accordance with protection as described above. Face-to-face sampling with the patient should be avoided as much as possible. Recommended precautions include having the patient take a seated position with the health worker standing to the patient's side, staggered head distance, standing upwind of the patient, and securing the patient's head with one hand while using the other hand for sampling (33). It is also critical for providers in generally uninvolved departments to pay attention to patients with fever, respiratory symptoms, and appropriate epidemiological history, as these may represent unscreened cases.

## Psychological Support for Healthcare Workers

As seen in previous epidemics, healthcare workers may have a higher risk of developing psychological issues; they may experience fear of contagion and spreading the virus to their families, friends, or colleagues (34). Those with previous first-line care experience, such as during the SARS epidemics, may also be at higher risk of post-traumatic stress symptoms. This could trigger common mental disorders, including anxiety and depressive disorders, and posttraumatic stress disorder that requires psychological intervention. However, further research is necessary to better assess the short- and long-term psychological consequences of this epidemic on healthcare workers.

It is important that hospital administrators be cognizant of the additional stressors faced by healthcare workers, provide appropriate training and protective equipment, manage work hours carefully, and implement quality of life measures to minimize burnout and risk of psychological consequences. In addition, safe communication channels between healthcare workers and their families should be set up, and psychological treatment plans, progress reports, and health status updates should be given to them and their families (35).

## Treatment

There are currently no specific antiviral drugs to treat SARS-CoV-2 infection. The treatment mainly includes supportive care including supplemental oxygen, symptom directed therapy, and the reduction of complications. Disease directed treatments that have been tried includes antivirals such a remdesivir and lopinavir/ritonavir, drugs such as chloroquine phosphate and abidol, and Chinese traditional medicines (24). On the Chinese Clinical Trial Registry^1^, a total of 473 clinical trials are registered, evaluating a variety of different drugs and other treatment modalities. In the United States, the National Institutes of Health (NIH) has started a clinical trial utilizing the experimental broad-spectrum antiviral drug remdesivir, launched on February 21 (36). The use of corticosteroid treatment is currently controversial (37, 38). Vaccines are under development in several countries around the world, which should help to contain the spread of COVID-19 in the future.

## Conclusions

In our review, we analyze current information on the characteristics of SARS-CoV-2 infection, and provide implementable approaches to standardized screening, triage, personal protection protocols, and psychological support for healthcare workers that are relevant to a variety of healthcare settings. As is the case with any review of a rapidly evolving topic such as COVID-19, it is impossible to be fully current, as new studies and guidelines are published on a daily basis. Likewise, this is a descriptive review rather than a systematic review, which limits the strength of the overall conclusions that can be drawn from the included studies. However, we believe this review provides a coherent, widely applicable framework for addressing these issues; one that can be modified as new information and new discoveries continue to come to light. This is particularly necessary for the issues of psychological support and treatment, as at the current time there is not enough outcome data available to make true evidence-based recommendations. Finally, while we review best practice PPE use, specific PPE choices may require modification depending on shortages, overall access to health care resources, and specific application.

COVID-19 has spread rapidly and progressed quickly to a pandemic associated with substantial morbidity and mortality. However, through early patient identification and accurate triage, risk of widespread transmission can be greatly decreased. Special attention must be paid to minimizing the additional risks faced by healthcare workers in order to ensure that appropriate care can continue to be provided.

## Summary

Potential COVID-19 cases must be identified early to initiate proper triage and distinguish them from similar infections. Early identification, accurate triage, and standardized personal protection protocols can reduce the risk of cross infection. Containing disease spread will require protecting healthcare workers.

## Author Contributions

YS, XL, YZ, and CC contributions to conception and design, and analysis and interpretation of data, drafting the article, final approval of the version to be published. KZ, JL, and JX contributions to acquisition of data, analysis and interpretation of data, conception and design. EN contributions to revising it critically for important intellectual content. YH, GQ, and BC interpretation of data, revising it critically for important intellectual content. MH, SB, FT, JH, SD, BS, SP, DJ, PF, and CR contributions to conception, revising it critically for important intellectual content and final approval. All of them are in agreement to be accountable for all aspects of the work in ensuring that questions related to the accuracy or integrity of any part of the work are appropriately investigated and resolved.

## Conflict of Interest

JH reported having a patent for cardiac cell-based therapy. JH holds equity in Vestion Inc. and maintains a professional relationship with Vestion Inc. as a consultant and member of the Board of Directors and Scientific Advisory Board. JH is the Chief Scientific Officer, a compensated consultant and advisory board member for Longeveron and holds equity in Longeveron. JH is the co-inventor of intellectual property licensed to Longeveron. The remaining authors declare that the research was conducted in the absence of any commercial or financial relationships that could be construed as a potential conflict of interest.

## Footnotes

^1^*Chinese Cliniclal Trial Registry*. Available online at: http://www.chictr.org.cn/enindex.aspx.

## References

[1] Hui DS Azhar EI Madani TA Ntoumi F Kock R Dar O. The continuing 2019-nCoV epidemic threat of novel coronaviruses to global health–The latest 2019 novel coronavirus outbreak in Wuhan, China. Int J Infect Dis. (2020) 91:264–6. 10.1016/j.ijid.2020.01.009 31953166 PMC7128332

[2] Paules CI Marston HD Fauci AS Coronavirus infections-more than just the common cold. JAMA. (2020) 323:707–8. 10.1001/jama.2020.0757 31971553

[3] World Health Organization Coronavirus Disease 2019 (COVID-19) Situation Report−57. World Health Organization (2020). Available online at: https://www.who.int/docs/default-source/coronaviruse/situation-reports/20200317-sitrep-57-covid-19.pdf?sfvrsn=a26922f2_4 (accessed March 31, 2020).

[4] Surging news Infection of Over 3000 Medical Staff in Hubei. Surging news (2020). Available online at: https://mp.weixin.qq.com/s/mnWPipga4ApDdrAYTrM5qA (accessed March 31, 2020).

[5] World Wide Web Summary of New Crown Pneumonia Epidemic Situation and Prevention and Control Information (as of 22:00 on the 24th Feb, 2020). World Wide Web (2020). Available online at: https://china.huanqiu.com/article/9CaKrnKpzxc (accessed March 31, 2020).

[6] Hoehl S Rabenau H Berger A Kortenbusch M Cinatl J Bojkova D. Evidence of SARS-CoV-2 infection in returning travelers from Wuhan, China. N Engl J Med. (2020) 382:1278–80. 10.1056/NEJMc2001899 32069388 PMC7121749

[7] Zou L Ruan F Huang M Liang L Huang H Hong Z. SARS-CoV-2 viral load in upper respiratory specimens of infected patients. N Engl J Med. (2020) 382:1177–9. 10.1056/NEJMc2001737 32074444 PMC7121626

[8] Wang D Hu B Hu C Zhu F Liu X Zhang J Clinical characteristics of 138 hospitalized patients with 2019 novel coronavirus-infected pneumonia in Wuhan, China. JAMA. (2020) 323:1061–9. 10.1001/jama.2020.1585 PMC7042881 32031570

[9] McGoogan ZWJM Characteristics of and important lessons from the coronavirus disease 2019 (covid-19) outbreak in China. Summary of a report of 72,314 cases from the Chinese Center for Disease Control and Prevention. JAMA. (2020) 323:1239–42. 10.1001/jama.2020.2648 32091533

[10] Zhou P Yang X-L Wang X-G Hu B Zhang L Zhang W. A pneumonia outbreak associated with a new coronavirus of probable bat origin. Nature. (2020). 579:270–3. 10.1038/s41586-020-2012-7 32015507 PMC7095418

[11] Ji W Wang W Zhao X Zai J Li X Homologous recombination within the spike glycoprotein of the newly identified coronavirus may boost cross-species transmission from snake to human. J Med Virol. (2020) 92: 433–40. 10.1002/jmv.25682 31967321 PMC7138088

[12] Guo Q Li M Wang C Wang P Fang Z Tan J Host and infectivity prediction of Wuhan 2019 novel coronavirus using deep learning algorithm. bioRxiv. (2020). 10.1101/2020.01.21.914044

[13] Liu P Jiang J-Z Wan X-F Hua Y Wang X Hou F Are pangolins the intermediate host of the 2019 novel coronavirus (2019-nCoV)? bioRxiv. (2020). 10.1101/2020.02.18.954628 PMC7224457 32407364

[14] Wrapp D Wang N Corbett KS Goldsmith JA Hsieh C-L Abiona O. Cryo-EM structure of the 2019-nCoV spike in the prefusion conformation. Science. (2020) 367:1260–3. 10.1101/2020.02.11.944462 32075877 PMC7164637

[15] Yan R Zhang Y Li Y Xia L Guo Y Zhou Q. Structural basis for the recognition of the SARS-CoV-2 by full-length human ACE2. Science. (2020) 367:1444–8. 10.1101/2020.02.19.956946 32132184 PMC7164635

[16] Wang K Chen W Zhou Y-S Lian J-Q Zhang Z Du P SARS-CoV-2 invades host cells via a novel route: CD147-spike protein. bioRxiv. (2020). 10.1101/2020.03.14.988345

[17] Guan W-j Ni Z-y Hu Y Liang W-h Ou C-q He J-x. Clinical characteristics of coronavirus disease 2019 in China. N Engl J Med. (2020) 382:1708–20. 10.1056/NEJMoa2002032 32109013 PMC7092819

[18] Lu H Ai J Shen Y Li Y Li T Zhou X A descriptive study of the impact of diseases control and prevention on the epidemics dynamics and clinical features of SARS-CoV-2 outbreak in Shanghai, lessons learned for metropolis epidemics prevention. medRxiv. (2020). 10.1101/2020.02.19.20025031

[19] Deng W Bao L Gao H Xiang Z Qu Y Song Z Rhesus macaques can be effectively infected with SARS-CoV-2 via ocular conjunctival route. bioRxiv. (2020). 10.1101/2020.03.13.990036

[20] van Doremalen N Bushmaker T Morris D Holbrook MG Gamble A Williamson BN Aerosol and surface stability of HCoV-19 (SARS-CoV-2) compared to SARS-CoV-1. MedRxiv. (2020) 382:1564–7. 10.1101/2020.03.09.20033217 PMC7121658 32182409

[21] Holshue ML DeBolt C Lindquist S Lofy KH Wiesman J Bruce J. First case of 2019 novel coronavirus in the United States. N Engl J Med. (2020) 382:929–36. 10.1056/NEJMoa2001191 32004427 PMC7092802

[22] Der Tagfsspiegel Wir müssen uns auf eine Pandemie einstellen(2020-02-13). Der Tagfsspiegel (2020). Available online at: https://www.tagesspiegel.de/wissen/deutscher-coronavirus-experte-wir-muessen-uns-auf-eine-pandemie-einstellen/25542906.html (accessed March 31, 2020).

[23] Chen H Guo J Wang C Luo F Yu X Zhang W. Clinical characteristics and intrauterine vertical transmission potential of COVID-19 infection in nine pregnant women: a retrospective review of medical records. Lancet. (2020) 395:809–15. 10.1016/S0140-6736(20)30360-3 32151335 PMC7159281

[24] Huang C Wang Y Li X Ren L Zhau J Hu Y. Clinical features of patients infected with 2019 novel coronavirus in Wuhan, China. Lancet. (2020) 395:497–506. 10.1016/S0140-6736(20)30183-5 31986264 PMC7159299

[25] Chen N Zhou M Dong X Qu J Gong F Han Y. Epidemiological and clinical characteristics of 99 cases of 2019 novel coronavirus pneumonia in Wuhan, China: a descriptive study. Lancet. (2020) 395:507–13. 10.1016/S0140-6736(20)30211-7 32007143 PMC7135076

[26] Yang X Yu Y Xu J Shu H Xia J Liu H. Clinical course and outcomes of critically ill patients with SARS-CoV-2 pneumonia in Wuhan, China: a single-centered, retrospective, observational study. Lancet Respiratory Med. (2020). 10.1016/S2213-2600(20)30079-5 32105632 PMC7102538

[27] Liu Y Sun W Li J Chen L Wang Y Zhang L Clinical features and progression of acute respiratory distress syndrome in coronavirus disease 2019. medRxiv. (2020). 10.1101/2020.02.17.20024166

[28] Del Rio C Malani PN COVID-19-new insights on a rapidly changing epidemic. JAMA. (2020) 23:1339–40. 10.1001/jama.2020.3072 32108857

[29] Zhang J Zhou L Yang Y Peng W Wang W Chen X. Therapeutic and triage strategies for 2019 novel coronavirus disease in fever clinics. Lancet Respiratory Med. (2020) 8:e11–2. 10.1016/S2213-2600(20)30071-0 32061335 PMC7159020

[30] Centers for Disease Control and Prevention (CDC). About Coronavirus Disease 2019 (COVID-19). (2020). Available online at: https://www.cdc.gov/coronavirus/2019-ncov/about/ (accessed March 31, 2020).

[31] World Health Organization Coronavirus Disease (Covid-19) Outbreak: Rights, Roles And Responsibilities Of Health Workers, Including Key Considerations For Occupational Safety And Health. World Health Organization (WHO). (2020). Available online at: https://www.who.int/docs/default-source/coronaviruse/who-rights-roles-respon-hw-covid-19.pdf?sfvrsn=bcabd401_0 (accessed March 31, 2020).

[32] Centers for disease control and prevention Guidance on Personal Protective Equipment (PPE) To Be Used By Healthcare Workers during Management of Patients with Confirmed Ebola or Persons under Investigation (PUIs) for Ebola who are Clinically Unstable or Have Bleeding, Vomiting, or Diarrhea in U.S. Hospitals, Including Procedures for Donning and Doffing PPE. Centers for disease control and prevention. (2020). Available online at: https://www.cdc.gov/vhf/ebola/healthcare-us/ppe/guidance.html (accessed March 31, 2020).

[33] Chinese Academy of Traditional Chinese Medicine Otolaryngology Branch Experts in Diagnosis and Treatment of Otolaryngology in Traditional Chinese Medicine (Integrated Chinese and Western Medicine) During the Outbreak of New Coronavirus Pneumonia. Chinese Academy of Traditional Chinese Medicine Otolaryngology Branch. (2020). Available online at: https://mp.weixin.qq.com/s/1xoWmbVLDmjn6EUag4EqJg (accessed March 31, 2020).

[34] Verma S Mythily S Chan YH Deslypere JP Teo EK Chong SA. Post-SARS psychological morbidity and stigma among general practitioners and traditional Chinese medicine practitioners in Singapore. Ann Acad Med. (2004) 33:743–8. 10.1097/00000441-200411000-00012 15608831

[35] Xiang Y-T Yang Y Li W Zhang L Zhang Q Cheung T. Timely mental health care for the 2019 novel coronavirus outbreak is urgently needed. Lancet Psychiatry. (2020) 7:228–9. 10.1016/S2215-0366(20)30046-8 32032543 PMC7128153

[36] ClinicalTrials.gov. Adaptive COVID-19 Treatment Trial (ACTT). ClinicalTrials.gov (posted on February 21, 2020). Available online at: https://clinicaltrials.gov/ct2/show/NCT04280705?cond=2019-nCoV&cntry=US&draw=2&rank=2 (accessed February 21, 2020).

[37] Shang L Zhao J Hu Y Du R Cao B. On the use of corticosteroids for 2019-nCoV pneumonia. Lancet. (2020) 395:683–4. 10.1016/S0140-6736(20)30361-5 32122468 PMC7159292

[38] Russell CD Millar JE Baillie JK Clinical evidence does not support corticosteroid treatment for 2019-nCoV lung injury. Lancet. (2020) 395:473–5. 10.1016/S0140-6736(20)30317-2 32043983 PMC7134694

